# The Stress of the Street for Patients With Persecutory Delusions: A Test of the Symptomatic and Psychological Effects of Going Outside Into a Busy Urban Area

**DOI:** 10.1093/schbul/sbu173

**Published:** 2014-12-20

**Authors:** Daniel Freeman, Richard Emsley, Graham Dunn, David Fowler, Paul Bebbington, Elizabeth Kuipers, Suzanne Jolley, Helen Waller, Amy Hardy, Philippa Garety

**Affiliations:** ^1^Department of Psychiatry, University of Oxford, Oxford, UK;; ^2^Centre for Biostatistics, Institute of Population Health, University of Manchester, Manchester Academic Health Science Centre, Manchester, UK;; ^3^School of Psychology, University of Sussex, Sussex, UK;; ^4^Department of Psychiatry, Faculty of Brain Sciences, University College London, UK;; ^5^Department of Psychology, King’s College London, Institute of Psychiatry, Psychology and Neuroscience, London, UK;; ^6^BRC of the South London and Maudsley NHS Foundation Trust, London UK

**Keywords:** delusions, paranoia, urbanicity, anxiety, depression

## Abstract

**Background:**

For many patients with persecutory delusions, leaving home and going into crowded streets is a key clinical problem. In this study we aimed to inform treatment development by determining the psychological mechanisms whereby busy urban environments increase paranoia. In a randomized design with prespecified mediation analysis, we compared the effects on patients of going outside into a busy social environment with staying inside.

**Methods:**

Fifty-nine patients with current persecutory delusions, in the context of nonaffective psychosis, reporting fears when going outside were assessed on factors from a cognitive model of paranoia. They were then randomized either to enter a busy local shopping street or to complete a neutral task indoors. They were then reassessed on the measures.

**Results:**

Compared with staying inside, the street exposure condition resulted in significant increases in paranoia, voices, anxiety, negative beliefs about the self, and negative beliefs about others. There was also a decrease in positive thoughts about the self. There was no alteration in reasoning processes. There were indications that the increase in paranoia was partially mediated by increases in anxiety (45%), depression (38%), and negative beliefs about others (45%).

**Conclusions:**

We found that increases in negative affect may form an important route by which social exposure in urban environments triggers paranoid thoughts. The study provides an illustration of how an experimental approach can be applied to help understand a specific difficulty for patients with psychosis. In future studies the effects of specific elements of the social environment could be tested.

## Introduction

In many patients with persecutory delusions, leaving their homes triggers paranoid thoughts. Being in busy, noisy places, surrounded by other people can be especially difficult. This leads to avoidance and, often, highly restricted lifestyles. In order to develop a precisely targeted treatment for this important clinical problem, the key mechanisms by which paranoia is caused in these situations need to be identified. Such understanding may also have more general theoretical significance in offering an explanation of the well-established finding that psychotic experiences are more common in those living in urban than in rural settings (eg, McGrath et al^1^, March et al^2^, Vassos et al^3^, Heinz et al^4^). We therefore set out to determine the immediate effects of entering a busy urban environment in patients with persecutory delusions.

A reasonable starting point is the assumption that fluctuations in delusional ideation—as triggered, eg, by going outside—are understandable in terms of activation of the mechanisms underlying delusions. We therefore used our cognitive model of persecutory delusions to understand the immediate effects of going into a busy urban environment.^5^ In this model, delusions are held to arise from an interaction of anomalous internal experiences, negative affect, and reasoning biases. It is hypothesized that individuals experience a changed and confusing anomalous internal state (eg, perceptual disturbances, unexplained arousal, or hallucinations occur). An explanation is needed for this ambiguous, but potentially threatening, event. Importantly, a negative affective state substantially raises the risk of a threatening paranoid interpretation. This may act through a number of routes. Anxiety provides the threat theme of paranoid thoughts, due to threat anticipation and a bias toward negative interpretations of ambiguous events. Paranoid fears also build upon negative views of the self, since the person is likely to feel inferior and hence apart and vulnerable. The effects will be enhanced by self-consciousness, an attentional focus on the self, increasing the sense of the self as a target. All these affective biases may of course arise from past experiences of real threat from others. The fears reach a delusional level of conviction when reasoning biases, such as belief inflexibility and jumping to conclusions (JTC), are present.

Drawing upon this model, it was predicted that going outside principally induces paranoia via the generation of negative affect. This is consistent with personal accounts of paranoia (eg, Adam^6^), and the hypothesis that urban environments are a stressor that engenders social defeat.^7^ Going outside is likely to cause patients to experience stress and hence the typical stress responses of anxiety and low mood. This will trigger a number of affective psychological processes such as threat anticipation, negative interpretations of events, negative thoughts about the self, and self-consciousness. In essence, there are changes in the contents of consciousness and the style of information processing that will raise the likelihood of paranoid ideation occurring. Principally, threat cognitions will come to mind, the self will be perceived as more vulnerable, and the focus of attention will be on danger.

In our pilot study 30 patients with persecutory delusions were randomized, either to going outside to buy a newspaper in a local shop in a busy street or to a relaxation task.^8^ Afterward, they completed measures of paranoia, affect, and reasoning. It was found that going outside led to an increase in paranoia, anxiety, negative beliefs about others, and JTC. Reasons for the increased paranoia were inferred but not tested. The current study improves on this pilot in a number of ways: a larger sample; a much more extensive assessment battery; and assessments carried out before and after the randomization condition, enabling tests of mediation. Moreover, the control condition was a neutral task (that was typical of inside activity), rather than relaxation, in order to specify the changes directly attributable to going outside. We hypothesized that social exposure would increase: paranoia, anxiety, data-gathering biases, and cognitive biases associated with anxiety (eg, threat anticipation, bias to interpret ambiguity as threat, self-focus). Furthermore, changes in paranoia would be mediated by the changes in the emotional and cognitive biases. The focus was on determining the psychological consequences and responsible mechanisms of the total effect of going outside into a busy social environment for patients with persecutory delusions, and not on dissecting the different contributions of the many and varied elements of the environment. The real clinical question of the in toto effects of going outside for patients was the target.

## Methods

### Participants

Sixty-one patients with persecutory delusions were recruited from 6 mental health sites: South London and Maudsley NHS Foundation Trust; Barnet, Enfield and Haringey Mental Health Trust; Camden and Islington Mental Health Foundation Trust; Oxleas NHS Foundation Trust; Central and North West London NHS Foundation Trust; and North East London NHS Foundation Trust.

The inclusion criteria were

- age 18–65
- a current persecutory delusion as defined by Freeman and Garety^10^
- the delusion occurred in the context of nonaffective psychosis (ICD-10, F2)
- the delusion had persisted for at least the last 3 months
- the belief was held with at least 50% conviction
- the belief was distressing
- the person experienced paranoia when going outside

The exclusion criteria were

- a primary diagnosis of alcohol or drug dependency
- presence of an organic syndrome
- learning disability
- insufficient grasp of English to complete the measures

Two patients did not attend appointments offered to complete the randomization condition.

### Assessments

#### Paranoia Measures

##### State Paranoia

Six Visual Analog Scales (VAS) were used to assess state paranoia: (1) I am being deliberately harmed or upset; (2) I am being followed; (3) There is a conspiracy against me; (4) I am being persecuted; (5) I am being laughed at behind my back; (6) I am feeling under threat. For each item, participants rated how they were feeling “right now” from 0 (Not at all) to 100 (Totally). There was also an additional VAS for how strongly the person currently believed the persecutory delusion, rated from 0 (“Believe not at all”) to 100 (“Believe absolutely”).

##### State Social Paranoia Scale

^9^ This was completed in relation to the last 15 min. It comprises 10 persecutory items (eg, “Someone stared at me in order to upset me”; “Someone was trying to isolate me”; “Someone had it in for me”; “Someone was trying to make me distressed”), each rated on a 5-point scale. Higher scores on the scale indicate greater levels of persecutory thinking. The State Social Paranoia Scale (SSPS) has excellent internal reliability, adequate test-retest reliability, and clear convergent validity with interviewer and self-report measures.

##### Schizotypal Symptoms Inventory—Paranoia

^11^ This was completed in relation to how the person felt “right now.” It has 7 items assessing paranoid thinking (eg, “I am sure I am being talked about behind my back” “Do you often feel that other people have it in for you?”), each rated on a 5-point scale (“not at all” to “all of the time”). Higher scores indicate greater levels of paranoia. The Schizotypal Symptoms Inventory (SSI) has excellent internal reliability and convergent validity and good test-test reliability.

#### Other Psychosis Measures

##### Hallucinations VAS

The occurrence of hallucinations was assessed on VAS assessing the frequency (“How much of the time does it occur?”) and associated distress (“How much does it upset you?”), rated on 0–100 scales. Higher scores indicate greater hallucinatory experience. These were only included by the subgroup of patients who experienced hallucinations.

##### Scale for the Assessment of Positive Symptoms,^12^ and Scale for the Assessment of Negative Symptoms^13^

The Scale for the Assessment of Positive Symptoms (SAPS) is a 35-item, 6-point (0–5) rating instrument for the assessment of the positive symptoms of psychosis. The Scale for the Assessment of Negative Symptoms (SANS) is a 25-item, 6-point (0–5) rating instrument for the assessment of the negative symptoms of psychosis. Both instruments were used only at the baseline assessment. The symptoms identified were rated over the preceding month. Higher scores indicate greater symptom levels.

#### Affective Measures

##### Anxiety and Depression VAS

Participants were asked to rate “How anxious are you feeling?” and “How depressed are you feeling?” from 0 (not at all) to 100 (totally).

##### Brief Core Schema Scales

^14^ The Brief Core Schema Scales (BCSS), developed with nonclinical and psychosis groups, has 24 items assessing negative and positive beliefs about the self and others each rated on a 5-point scale (0–4). Four subscale scales are obtained: negative self (eg, “I am unloved,” “I am worthless”), positive self (eg, “I am respected,” “I am valuable”), negative other (eg, “Other people are hostile, Other people are harsh”), positive other (eg, “Other people are fair,” “Other people are good”). Higher scores indicate greater endorsement of items. The scale has good internal reliability, test-retest reliability, and convergent validity.

##### Self-Focus

^15^ Three VAS assessed current focus of attention (“Right now my attention is focused on my inner thoughts and feelings,” “Right now my attention is focused on how I appear to others,” “Right now my attention is focussed on my surroundings”). Each was rated on a 0 (“not at all”) to 100 (“Totally”) scale.

##### Threat Anticipation

^15^ The format was derived from previous studies (ref.^16^). Participants had to rate how likely 5 listed, mildly negative, events were to happen over the next 2 years to themselves (on a scale of 0 “not at all likely” to 7 “very likely”). We used 5 mild negative events that were not obviously paranoia-related (“Your physical health deteriorates,” “You will find it hard to express yourself with others,” “You have too many responsibilities to manage,” “You have an accident,” “You cannot manage your finances”). A higher total score indicates higher estimates of likelihood.

##### Interpretation of Ambiguity

^17^ In this task 10 ambiguous scenarios are presented to participants, and respondents answer yes or no to a possible explanation. For example: *You go to a party at a club. While dancing, you spot an old friend not far away and call out. They do not reply, and after a moment, turn and leave the dance floor, heading for the bar. You don’t call out again because it is too noisy. Was your friend ignoring you in the club? (Yes* */* *No).* Other scenarios concern, eg, giving a presentation, doing a DIY project, and first impressions. Higher scores indicate greater endorsement of negative interpretations of the ambiguous events.

#### Reasoning Measures

##### The Beads Task

^18^ Two computerized versions of the probabilistic reasoning (Beads) task, with 85:15 (easy) and 60:40 (difficult) task ratios, were used to assess JTC. For example, for the easy version of the task, one jar had 85 orange beads and 15 black beads and the other jar had 15 orange beads and 85 black beads. Participants were shown pictures of the 2 jars and told that one of the jars would be selected at random by the computer and that beads would be drawn from and replaced in the selected jar. After each bead was drawn, participants were asked if they would like to see more beads or if they could say, with certainty, from which of the jars the beads were being drawn. Once a bead had been drawn, it was shown at the bottom of the screen thereby providing a memory aid. The key variable was the number of beads requested by the participant before making a decision (draws to decision). JTC was classified as requesting 2 or fewer beads.

##### Maudsley Assessment of Delusions

^19^ Two of the Maudsley Assessment of Delusions Scale (MADS) items are used to measure aspects of belief flexibility (the possibility of being mistaken, and the reaction to hypothetical contradiction). The evidence for the delusion cited by participants is sensitively discussed, and they are asked whether it is at all possible for them to be mistaken about their delusional belief. The interviewer then asks how they would react in a hypothetical situation if some new evidence were to be generated which contradicts the delusion. If they report that this would alter in any way their level of belief, this is recorded as belief flexibility.

##### The Explanations of Experiences Measure

^20^ The Explanations of Experiences (EoE) measure is a structured interview designed to assess whether people can envisage alternative explanations for the evidence cited for their delusion. Once the evidence for the delusion is established, they are asked “Can you think of any other explanations for the experiences that you have described? Are there any other reasons — other than [the delusional belief] — that could possibly account for these experiences even if you think they are very unlikely?” The generation of any alternative explanation is also taken as a measure of belief flexibility.

##### Weschler Test of Adult Reading

^21^ Premorbid IQ was estimated at baseline using the Weschler Test of Adult Reading (WTAR), which comprises pronouncing 50 irregularly spelled words.

### Design

The design involved random allocation between 2 experimental conditions: street exposure or a neutral control condition. Patients attended 2 meetings to carry out the research. In the first, they completed the nonrepeated measures (ie, SAPS, SANS, WTAR). The actual experiment, including the randomization conditions, was carried out at the second testing session. Randomization to condition using an online system was carried out during this second session, ie, after all the baseline assessments had been completed. A sealed envelope with the randomization condition was only opened once all the premanipulation assessments had been completed during the second meeting.

For the street exposure condition, the preference was to use identified shopping streets in areas of relative deprivation near each mental health treatment center, but we also used shopping streets near to a person’s home when he or she was unable to come to clinic settings. Identified streets were required to have people walking down them during the day, to have a bus stop with queues to walk past, and a supermarket shop. There was variability in the degree to which patients were familiar with the streets. The street exposure condition was designed to last approximately 10 min, and to take place around mid-day. Participants were given money to go to an identified local shop to make a purchase for themselves (eg, milk). The research assessor walked part of the way and then the patient continued unaccompanied. For the control condition, patients sat in a room with the research assessor and watched mildly humorous television clips for approximately 10 min. After the randomization condition was complete, the assessments were repeated. The main paranoia and emotional self-report measures were completed out on the street with the assessor for the exposure condition group. The second meeting could be rescheduled if the weather prevented the street exposure from taking place.

The research protocol was approved by an NHS research ethics committee, and all participants provided written informed consent. The current study was 1 of 2 separate but linked studies, employing randomized experimental designs to test hypothesized mechanisms of change in delusions, using common measures but separate patient samples and hypotheses (see Garety et al^22^).

### Analysis

All analyses were carried out using Stata version 13.1.^23^ First a single standardized latent paranoia outcome measure was constructed, using the SSPS total, the SSI paranoia subtotal, the 6 VAS, and the conviction rating. Factor loadings for the latent paranoia variable were derived from the premanipulation assessment, and then used to calculate the factor scores at the postrandomization condition assessment. In a conventional intention-to-treat (ITT) approach, ANCOVA was used to evaluate the effect of the randomization condition on the outcome (paranoia) and, separately, the putative mediators (eg, anxiety) as dependent variables. We allowed for center and the baseline measures of the outcome or mediator (as appropriate) as covariates in these models.

Mediation analysis was performed using the causal mediation approach outlined in Valeri and VanderWeele^24^ to investigate direct and indirect effects of the experimental manipulation on paranoia. In addition to the previous ITT models, this involved regressing paranoia on the randomized condition and the mediators in the same linear model. The effect of randomized condition on the mediator and the effect of the mediator on paranoia are multiplied to estimate the indirect effect, assuming there is no interaction between randomized condition and mediator on outcome. The SEs of the direct and indirect effects were generated using Monte Carlo bootstrapping with 200 replications. The proportion mediated was calculated as the indirect effect divided by the total effect. Since a variable can only be a mediator if there is a significant effect of randomized condition on the mediator, mediation analysis was only performed when there was a significant ITT effect on the mediators.

We performed the mediation analysis with and without adjustment for baseline covariates in all 3 models. Estimates of the direct and indirect effects can be biased, even in randomized trials, when there are unmeasured confounders between the mediator and outcome.^25^ By including baseline measures of the outcome and mediators in the regression models, we attempt to control for these as potential confounders in order to add robustness to our analysis. The results presented here are of complete cases, so that patients with missing outcomes or mediator values are not included in the analysis; we indicate the numbers included in our results. This approach assumes that, conditional on the baseline covariates and randomization, the missing outcomes and mediators are missing at random.

## Results

### Basic Demographic and Clinical Data

The demographic and basic clinical data for the patients who completed the randomization conditions are reported in table 1. Diagnoses were derived using OPCRIT (Operational CRITeria) from the Schedules for Clinical Assessment in Neuropsychiatry (SCAN) interviews,^26^ with schizophrenia the most common diagnosis received by participants.

**Table 1.** Summary of Demographic and Clinical Data

	Street Exposure (*n* = 28)	Control (*n* = 31)
Age in years (SD)	43.8 (10.0)	42.9 (11.5)
Male, female (*n*)	14, 14	21, 10
Ethnicity (*n*)		
White	18	16
Black (African, Caribbean, or British)	8	11
Asian	2	0
Other	0	3
Diagnosis (*n*)		
Schizophrenia	22	26
Delusional disorder	3	2
Schizoaffective disorder	1	2
Length of illness in years (SD)	15.2 (10.9)	16.5 (11.4)
Chlorpromazine equivalent dose (SD)	496.6 (502)	480.7 (366.6)
Total SAPS mean global rating (SD)	8.0 (2.8)	7.4 (2.7)
Total SANS mean global rating (SD)	6.4 (3.1)	5.8 (3.3)
Had experienced an assault with weapon (*n*)	12	15
Had experienced a serious assault without weapon (*n*)	12	14
IQ (SD)	93.9 (16.6)	94.7 (19.9)

*Note*: SANS, Scale for the Assessment of Negative Symptoms; SAPS, Scale for the Assessment of Positive Symptoms.

### Primary ITT Analysis

Table 2 shows the scores on the assessment measures pre- and postrandomization. Table 3 shows ITT effects on the outcome paranoia variable and the potential mediator variables, adjusting for prerandomization values of each measure and the recruitment center. The effect is the adjusted difference in the outcome means of the street exposure group compared with the control group after allowing for covariates. It can be seen that going out into the street led to a significant increase in paranoia, compared with remaining inside. Not all patients completed all the paranoia measures, but it is notable that results were also significant for individual paranoia measures such as the SSPS, *n* = 54, effect = 5.98, SE = 2.28, *P* = .012, and the SSI paranoia items, *n* = 57, effect = 2.14, SE = 0.77, *P* = .007. The street exposure also led to significant increases in anxiety, depression, negative self beliefs, negative other beliefs, and hallucinations. There was a significant reduction in positive beliefs about the self.

**Table 2.** Pre- and Postrandomization Condition Scores for Each Outcome and Mediator Measure

Measure	Time	Experimental Group				Control Group			
		Mean	SD	Range	*N*	Mean	SD	Range	*N*
Paranoia outcomes									
Paranoia latent variable	Pre	0.27	1.06	−1.14, 1.73	25	−0.26	0.78	−1.12, 1.94	28
	Post	0.44	1.22	−1.00, 2.59	26	−0.30	0.70	−0.99, 1.44	28
I am being deliberately harmed or upset	Pre	30.39	38.05	0–100	28	18	29.63	0–100	31
	Post	35.89	38.47	0–100	28	7	17.69	0–65	30
There is a conspiracy against me	Pre	40.39	44.42	0–100	28	30.90	35.09	0–100	31
	Post	39.82	39.82	0–100	28	27.87	38.08	0–100	30
I am being followed	Pre	32.89	38.06	0–100	28	19.94	28.82	0–100	31
	Post	31.96	31.95	0–100	28	20.33	33.50	0–100	30
I am being persecuted	Pre	46.46	42.62	0–100	28	26.03	33.10	0–100	31
	Post	35	40.69	0–100	28	21.17	32.61	0–100	30
I am being laughed at behind my back	Pre	36.71	32.88	0–100	28	22.10	27.14	0–100	30
	Post	32.5	35.08	0–100	28	12.79	20.75	0–75	29
Feeling under threat	Pre	28.31	34.40	0–100	26	22.16	28.69	0–100	31
	Post	38.65	37.75	0–100	26	16.73	22.90	0–75	30
SSPS—total	Pre	19.76	12.50	10–50	25	14.62	8.79	10–45	29
	Post	19.68	12.22	10–50	28	11.68	5.79	10–42	31
SPQ—total	Pre	38.11	17.90	3–68	28	33.50	12.72	7–57	30
	Post	40.18	20.97	2–80	28	30.00	14.46	4–55	30
SPQ—paranoia	Pre	13.57	7.08	0–24	28	11.17	4.93	1–24	30
	Post	14.07	7.53	0–24	28	9.37	5.73	0–22	30
Voices									
Hallucinations	Pre	323.24	96.68	100–480	21	324.67	84.72	155–500	21
	Post	353.71	103.08	100–500	21	314.10	81.46	165–450	21
Distress	Pre	61.19	33.28	0–100	21	53.00	31.89	0–100	21
	Post	67.81	30.01	0–100	21	51.29	27.50	10–100	21
Affective mediators									
Anxiety	Pre	39.68	27.97	0–100	28	33.55	29.51	0–100	31
	Post	52.68	35.62	0–100	28	23.43	26.94	0–100	30
Depression	Pre	36.04	28.71	0–95	28	26.45	23.24	0–75	31
	Post	35.43	32.05	0–100	28	16.27	21.78	0–75	30
BCSS—negative self	Pre	6.79	5.48	0–18	28	7.10	5.49	0–21	31
	Post	7.81	5.82	0–24	27	6.23	5.71	0–20	31
BCSS—positive self	Pre	11.79	7.54	0–24	28	9.10	5.80	0–20	31
	Post	10.07	7.38	0–23	27	10.06	6.13	0–22	31
BCSS—negative other	Pre	11.18	7.21	0–24	28	9.81	6.09	0–24	31
	Post	13.19	7.84	0–24	27	9.10	7.11	0–24	31
BCSS—positive other	Pre	9.11	6.25	0–22	28	10.23	6.24	0–24	31
	Post	7.63	5.65	0–17	27	10.37	6.42	0–24	30
Self focus—inner thoughts	Pre	55.29	29.45	0–100	28	51.80	29.64	0–100	30
	Post	55.37	31.22	0–100	27	53.35	31.72	0–100	31
Self focus—how I appear	Pre	40.29	30.18	0–100	28	36.23	26.85	0–75	30
	Post	33.67	31.46	0–100	27	31.45	27.90	0–90	31
Self focus—surroundings	Pre	43.11	32.03	0–100	28	37.83	26.67	0–100	30
	Post	44.93	32.65	0–100	27	40.58	28.33	0–100	31
Threat anticipation	Pre	18.85	6.48	7–30	27	17.58	5.70	6–28	31
	Post	19.11	5.42	8–28	27	16.84	6.03	5–26	31
Interpretation bias	Pre	4.30	2.38	0–10	27	4.39	2.49	0–9	31
	Post	5.35	2.43	2–10	26	4.63	2.50	1–10	30
Reasoning mediators									
Probability of being mistaken (%)	Pre	20.71	23.68	0–80	28	25.13	29.91	0–100	31
	Post	10.54	16.74	0–50	28	18.83	23.88	0–80	30
Number of beads—85/15	Pre	3.67	4.16	1–19	27	3.97	4.61	1–20	31
	Post	4.26	4.71	1–19	27	5.06	5.62	1–20	31
Number of beads—60/40	Pre	6.19	6.04	1–20	27	6.16	5.60	1–20	31
	Post	6.19	5.73	1–20	27	7.00	5.84	1–20	31

*Note*: BCSS, Brief Core Schema Scale; SSPS, State Social Paranoia Scale.

**Table 3.** Effect of Street Exposure Compared With the Control Group

Measure	Effect	SE	*P*	95% CI	*N*
Paranoia					
Paranoia outcome variable	0.37	0.17	.037	0.02, 0.71	51
Affective mediators					
Anxiety	27.48	8.04	.001	11.36, 43.61	58
Depression	12.98	5.71	.027	1.54, 24.43	58
BCSS—negative self	1.68	0.76	.032	0.15, 3.21	58
BCSS—positive self	−2.09	0.98	.036	−4.04, −0.15	58
BCSS—negative other	2.82	0.99	.006	0.83, 4.81	58
BCSS—positive other	−1.84	1.36	.066	−3.80, 0.13	57
Self-focus—inner thoughts	−0.36	6.99	.959	−14.38, 13.65	57
Self-focus—how I appear	−2.06	6.45	.750	−15.00, 10.87	57
Self-focus—surroundings	1.95	7.45	.795	−13.00, 16.89	57
Threat anticipation	1.56	1.24	.212	−0.92, 4.04	58
Interpretation bias	0.79	0.50	.122	−0.22, 1.79	55
Reasoning mediators					
Probability of being mistaken—%	−7.19	4.23	.095	−15.67, 1.30	58
JTC—85/15	OR = 1.67	1.21	.483	0.40, 6.94	58
JTC—60/40	OR = 1.09	0.96	.918	0.20, 6.11	58
PM—yes/no	OR = 0.60	0.35	.390	0.19, 1.91	59
Alternative explanations	OR = 0.96	0.66	.957	0.25, 3.70	56
Hypothetical contradiction	OR = 1.15	0.72	.829	0.33, 3.95	59
Voices					
Hallucination frequency—VAS	43.15	17.82	0.020	7.08, 79.22	42
Distress—VAS	13.20	7.01	0.067	−1.00, 27.39	42

*Note*: BCSS, Brief Core Schema Scale; JTC, jumping to conclusions; PM, possibility of being mistaken; VAS, Visual Analog Scale.

### Mediation Analysis

The results for the mediation analysis for the increase in paranoia are shown in table 4. The analyses present results with and without adjustment for the pretest values for paranoia and all the putative mediators, together with recruitment center, as covariates. In the adjusted analysis, there was evidence of partial mediation (approximately 40%) by anxiety, negative beliefs about others, and depression, and, to a lesser extent (15%), negative beliefs about the self. The evidence of mediation was not statistically significant. In the unadjusted analysis, we observed larger total effects than the adjusted analysis, and found significant indirect effects through anxiety (*P* = .03) and depression (*P* = .02). In the subgroup with hallucinations, we also tested whether increasing hallucination frequency explained the increase in paranoia, but there was little evidence of mediation (*n* = 36, mediated effect = 0.02, SE = 0.05, *P* = .64, proportion mediated = 3.8%).

**Table 4.** Statistical Mediation Analysis for the Latent Paranoia Outcome at Postmeasure

Mediator	Total Effect Effect (Boot SE), *P*	Direct Effect Effect (Boot SE), *P*	Mediated Effect Effect (Boot SE), *P*	Proportion Mediated (%)	*n*
Anxiety	0.39 (0.16), .01	0.21 (0.16), .19	0.18 (0.11), .09	45.12	51
	0.74 (0.28), .01	0.29 (0.28), .30	0.45 (0.20), .03	60.30	54
Depression	0.39 (0.16), .01	0.24 (0.14), .09	0.15 (0.11), .18	37.59	51
	0.74 (0.28), .01	0.31 (0.23), .18	0.43 (0.19), .02	57.72	54
BCSS—negative self	0.39 (0.16), .01	0.33 (0.18), .06	0.06 (0.08), .48	15.00	51
	0.74 (0.28), .01	0.65 (0.27), .02	0.08 (0.11), .45	11.44	54
BCSS—positive self	0.39 (0.16), .01	0.40 (0.17), .02	−0.01 (0.05), .92	−1.39	51
	0.74 (0.28), .01	0.74 (0.28), .01	−0.00 (0.05). .96	−0.30	54
BCSS—negative other	0.39 (0.16), .01	0.22 (0.16), .17	0.18 (0.11), .13	44.82	51
	0.74 (0.28), .01	0.44 (0.25), .08	0.30 (0.20), .13	40.27	54

*Note*: Within each mediator, the top row shows the adjusted analysis, the bottom row shows the unadjusted analysis. BCSS, Brief Core Schema Scale.

## Discussion

In this study we took an experimental approach to understanding a key clinical problem for patients with persecutory delusions. In a randomized controlled design, symptom and mechanism measures were taken before and after going outside into a busy urban environment, and compared with those taken from patients who remained inside. This may be framed as a paranoia induction method with the aim of determining the underlying psychological mechanisms. As predicted, patients who went outside experienced increased paranoia compared with those who remained inside. It was also found that the street exposure condition was associated with increases in anxiety, depression, negative views about the self, negative views about others, and hallucinations, and a reduction in positive views of the self. There were, however, no differences in reasoning processes, either JTC or flexibility in relation to the delusional belief: this was a failure to replicate the result found in the pilot study.^8^ It was however consistent with patient studies that found JTC does not alter in response to mood induction processes (eg, So et al^27^, Freeman et al^28^). Overall, going outside was associated with significant changes in affect and related processing, but not in reasoning styles.

Our results indicated that the increase in paranoia was partially mediated by anxiety, depression, and negative schematic beliefs. The study had limited power to detect whether mediated effects were statistically significant, since our sample size had 80% power to detect only large indirect effects.^29^ However, the pattern of results is consistent with increases in affect explaining a reasonably large degree of the difficulties of urban exposure. Interestingly schematic beliefs may have accounted for changes in paranoia, while attentional focus and threat anticipation did not. This is consistent with the view that urban environments influence mental health through a process of social defeat,^7^ and the specific hypothesis that paranoia builds upon negative beliefs about the self.^30,31^ It indicates that interventions specifically targeted at helping patients with persecutory delusions to go outside (see Freeman et al^32^) may benefit from an explicit focus on views about the self and others. Surprisingly, an increase in hallucinations was not associated with an increase in paranoia: this is inconsistent with our clinical experience. This negative finding may perhaps be due to the fact that a fair proportion of the participants did not have hallucinations, and that the paranoia assessments were not specifically tied to hallucinatory experience.

A cognitive model of persecutory delusions^5,33^ was used to derive the study hypotheses, but what does the study tell us about the theory? It clearly supports the central tenet that affect is a key factor in severe paranoia. Beliefs about the self and others were the key psychological process identified, although this assumes there is comparability in sensitivity to change in the assessment of each affective variable. Variables such as threat anticipation may simply be harder to measure accurately in a questionnaire. It is also of note that the putative mediators covered a range of cognitive and emotional processes but that it would have been valuable to have assessed other potentially relevant affective mechanisms such as worry, safety behaviors, and interpersonal sensitivity. Nonetheless, we can be reasonably confident that reasoning processes such as JTC, which we have shown elsewhere have adequately sensitive measurement to show short-term change,^22^ were not altered by social exposure. It is plausible that different causal factors highlighted in the model come into action at different stages of delusional belief formation and maintenance.

The sort of experimental approach we took to paranoia has a number of limitations. There will have been variability in the social environments that the patients were exposed to, and in their familiarity with them. This is, we hope, compensated by the ecological validity and clinical relevance of the procedures. Perhaps more problematic is that a one-off exposure within a research protocol does not fully capture the reality for patients of everyday visits taken alone. In the experimental setting there is likely to be less exacerbation of paranoia, as patients take a degree of reassurance from the presence of the researchers; this is an additional constraint, together with the sample size, on the power of the study to detect significant effects. There may also be differences related to how long people have held their fears about going outside. We chose a neutral condition that was moderately engaging for patients, and likely to reflect actual activity indoors, but other activities might have different impacts on psychological processes.

The mediation analysis assumes that there are no unmeasured confounders between the mediator and outcome, but such confounding is possible since both variables are measured postrandomization. We attempted to control for some confounders by including baseline measures as covariates in the mediation analysis; however, we cannot rule out the presence of further unmeasured confounders influencing the results. Although the statistical analysis was consistent with the hypothesis that anxiety leads to paranoia, the reverse causal pathway cannot be ruled out since these measures were taken at the same measurement occasion; we cannot empirically demonstrate which change occurred first. We note the advice of Bullock and colleagues^34^ “to think of mediation analysis as a cumulative enterprise.” Therefore, building upon the current work, an interesting future causal test would be to use anxiety reduction before social exposure.

The study of the psychological effects of urban environments on patients with mental health problems is clearly at an early stage. Future studies could dissect the effects of different aspects of the environment, eg, between going outside into places with and without other people, or between noisy and quiet social situations, or between familiar and unfamiliar locations. Use of virtual reality scenarios may be helpful in this work.^35^ Factors that moderate effects, such as previous experience of physical attack, or social support, or working memory impairments, could be tested in larger studies. The effects of social environments on patients compared to those without clinical disorders would be of interest. The central clinical question raised in the current study can lead to a substantial research program.

## Funding

This study was supported by a project grant from the Wellcome Trust (ref. 085396). D.F. is supported by a Medical Research Council (MRC) Senior Clinical Fellowship (G0902308). P.G. and E.K. acknowledge support of the NIHR BRC of the South London and Maudsley NHS Foundation Trust. R.E. and G.D. received research funding from the UK Medical Research Council (G0900678) and R.E. through a Medical Research Council Career Development Award (G0802418).
